# Reduced Plasma Extracellular Vesicle CD5L Content in Patients With Acute-On-Chronic Liver Failure: Interplay With Specialized Pro-Resolving Lipid Mediators

**DOI:** 10.3389/fimmu.2022.842996

**Published:** 2022-03-07

**Authors:** María Belen Sánchez-Rodríguez, Érica Téllez, Mireia Casulleras, Francesc E. Borràs, Vicente Arroyo, Joan Clària, Maria-Rosa Sarrias

**Affiliations:** ^1^ Biochemistry and Molecular Genetics Service, Hospital Clínic-Institut d'Investigacions Biomèdiques August Pi i Sunyer (IDIBAPS), Barcelona, Spain; ^2^ Innate Immunity Lab, Health Research Institute Germans Trias i Pujol (IGTP), Badalona, Spain; ^3^ Innovation in VEsicles and Cells for Application in Therapy (IVECAT), Health Research Institute Germans Trias i Pujol (IGTP), Badalona, Spain; ^4^ European Foundation for the Study of Chronic Liver Failure (EF Clif) and Grifols Chair, Barcelona, Spain; ^5^ Department of Biomedical Sciences, Universitat de Barcelona, Barcelona, Spain; ^6^ CIBER (Center of Biomedical Research in Network) of Hepatic and Digestive Diseases (CIBERehd), Barcelona, Spain

**Keywords:** macrophages, cirrhosis, resolution of inflammation, RvE1, lipid autacoids

## Abstract

Acute-on chronic liver failure (ACLF) is a syndrome that develops in patients with acutely decompensated cirrhosis (AD). It is characterized by a systemic hyperinflammatory state, leading to multiple organ failure. Our objective was to analyze macrophage anti-inflammatory protein CD5L in plasma extracellular vesicles (EVs) and assess its as yet unknown relationship with lipid mediators in ACLF. With this aim, EVs were purified by size exclusion chromatography from the plasma of healthy subjects (HS) (n=6) and patients with compensated cirrhosis (CC) (n=6), AD (n=11) and ACLF (n=11), which were defined as positive for CD9, CD5L and CD63 and their size, number, morphology and lipid mediator content were characterized by NTA, EM, and LC-MS/MS, respectively. Additionally, plasma CD5L was quantified by ELISA in 10 HS, 20 CC and 149 AD patients (69 ACLF). Moreover, macrophage CD5L expression and the biosynthesis of specialized lipid mediators (SPMs) were characterized *in vitro* in primary cells. Our results indicate that circulating EVs were significantly suppressed in cirrhosis, regardless of severity, and showed considerable alterations in CD5L and lipid mediator content as the disease progressed. In AD, levels of EV CD5L correlated best with those of the SPM RvE1. Analysis of total plasma supported these data and showed that, in ACLF, low CD5L levels were associated with circulatory (p<0.001), brain (p<0.008) and respiratory (p<0.05) failure (Mann-Whitney test). Functional studies in macrophages indicated a positive feedback loop between CD5L and RvE1 biosynthesis. In summary, we have determined a significant alteration of circulating EV contents in ACLF, with a loss of anti-inflammatory and pro-resolving molecules involved in the control of acute inflammation in this condition.

## Introduction

Cirrhosis is a progressive chronic liver disease characterized by extensive hepatic fibrosis, disruption of hepatic blood flow, portal hypertension and liver failure. During the evolution of the disease, compensated cirrhosis (CC) may lead to acutely decompensated cirrhosis (AD), involving the development of ascites, variceal hemorrhage and/or hepatic encephalopathy. AD is a risk factor for a fatal syndrome known as acute-on-chronic liver failure (ACLF) (1).

A hallmark of patients with cirrhosis and ACLF is systemic inflammation, which results in alterations of the number and function of circulating leukocyte components. In these patients, leukocytosis is a predictor of death and several features of unresolved inflammation and unremitting activation of immune cells (1, 2). Indeed, immune cells (i.e., polymorphonuclear leukocytes (PMNs), monocytes and T and B lymphocytes) are the main cellular players in systemic inflammation, and the activation of these cells is regulated by numerous factors, among them the protein CD5L (CD5-Like).

Produced mainly by macrophages, CD5L is a 40-kDa glycoprotein that plays diverse roles at the intersection between lipid homeostasis and the innate immune response and thus participates in many processes, including infection, atherosclerosis and cancer (3). In the context of inflammation, CD5L is emerging as a critical regulator of inflammation by inhibiting immune cell activation and preventing the secretion of pro-inflammatory cytokines by this cell population (3, 4).

Interestingly, this protein is enriched in extracellular vesicles (EVs) purified from plasma (5). The latter are cell-secreted nano-vesicles present in human fluids, and their content provides a molecular fingerprint of the releasing cell and, importantly, its status. Given that EVs are enriched in highly selected biomolecules, which may comprise only a very small proportion of the total content of body fluids, they are a potential source of biomarkers in human disease. This feature can facilitate the discovery of biomarkers expressed at relatively low levels that would be difficult to determine in whole fluids (6). Importantly, CD5L modulates the processing of polyunsaturated fatty acids (PUFAs) and the formation of bioactive lipid mediators derived from these molecules (7). In fact, EVs released from immune cells, especially monocytes, macrophages and neutrophils, carry and deliver PUFA-derived bioactive lipids, protect bioactive lipid mediators from degradation, and are a nidus for the biosynthesis of specialized pro-resolving mediators (SPMs) (8, 9). The latter are endogenous lipids that orchestrate the resolution of inflammation, a tightly regulated process whose dysregulation is commonly associated with uncontrolled inflammation (10).

The term SPM embraces a family of chemically and functionally distinct lipid mediators derived from PUFAs and that have dual roles as stop-signals for inflammation and activators of its resolution (10, 11). For instance, SPMs pave the way for monocyte differentiation into phagocytic macrophages, facilitating the removal of dead or dying cells and bacterial clearance, as well as enhancing phagocyte efflux from inflamed tissues to draining lymph nodes to facilitate resolution (10, 11). In broad terms, the resolution of inflammation is an actively regulated process governed by an array of mediators (i.e., SPMs) as diverse as those that initiate inflammation.

In this study, we hypothesized that EVs circulating in plasma have key immunomodulatory potential in the context of chronic liver disease (12). Accordingly, the deregulation or loss of the immunomodulatory EV cargo in patients with advanced stages of liver disease, such as AD and ACLF, could contribute to the systemic hyperinflammatory status of these subjects. To explore this hypothesis, here we examined the plasma EV content of CD5L and bioactive lipid mediators during chronic liver disease progression and identified a novel functional link between CD5L and the SPM resolvin E1 (RvE1), two immunomodulatory signaling pathways that are impaired in ACLF.

## Materials and Methods

### Patients and Biological Samples

The study used biobank plasma samples from 80 patients with AD cirrhosis without ACLF (a group hereafter called “AD”) and 69 patients with AD cirrhosis with ACLF (a group hereafter called “ACLF”) from the CANONIC study. The criteria for patient selection was based on availability of plasma samples at enrollment and intensive surveillance (clinical assessment) within the 28-day follow-up. The study also included 20 patients with compensated cirrhosis (a group hereafter called “CC”), who had clinically significant portal hypertension as indicated by presence of esophageal varices and/or high fibroscan liver stiffness, as well as 10 healthy subjects (HS) with matched sex and age. For the isolation of EVs, 6 plasma samples from HS, and pools of plasma samples (6 for CC, 11 for AD and 11 for ACLF) were analyzed. The pooling of plasma samples was needed to reach the volume required for EV extraction, and was performed at 4°C to preserve the quality of the samples. All studies involving human samples were conducted following the Declaration of Helsinki principles and current legislation on the confidentiality of personal data and were approved by the Human Ethics Committee of the Hospital Clínic of Barcelona and Hospital Germans Trias i Pujol.

### Human Monocyte-Derived Macrophage (HMDM) Cultures

PBMCs from 8 HS recruited at the Blood Bank of the Hospital Clínic of Barcelona were isolated from 20 mL of peripheral blood in EDTA tubes. Blood samples were centrifuged at 200 g for 10 min at room temperature (rt) to collect plasma, and sedimented cells were diluted with Dulbecco’s Phosphate-Buffered Saline without calcium and magnesium (DPBS^-/-^) up to a volume of 20 mL. Diluted blood was layered over 13.3 mL of Ficoll-Paque (GE Healthcare Life Sciences) and centrifuged at 500 g for 25 min. PBMCs were obtained from the mononuclear cell layer, incubated with pre-warmed ammonium-chloride-potassium lysis buffer for 10 min at rt to remove red blood cells, then centrifuged at 400 g for 5 min, and finally washed with DPBS^-/-^. PBMCs were counted and resuspended in RPMI 1640 medium containing penicillin (100 U/mL), streptomycin (100 U/mL) and L-glutamine (4 mM). They were seeded at a density of 5x10^6^ cells/well with RPMI medium in 24-well plates. For peripheral blood monocyte differentiation into macrophages, cells were allowed to attach at 37°C in a 5% CO_2_ atmosphere for 2 h and non-adherent cells were discarded while adherent ones were incubated with fresh RPMI medium supplemented with 10% fetal bovine serum (FBS). The medium was replaced with fresh RPMI with 10% FBS after 2-3 days. After 5-6 days of culture in these conditions, HMDMs were grown for one day with FBS-free RPMI medium and were incubated for 6 h at 37°C in a 5% CO_2_ atmosphere either with vehicle (0.007% ethanol) or 5S,12R,18R-trihydroxy-6Z,8E,10E,14Z,16E-eicosapentaenoic acid (RvE1) (10 nM) (Cayman Chemical, Ann Arbor, MI). After 2 h of incubation, lipopolysaccharide serotype O26:B6 (LPS) (Sigma-Aldrich, St Louis, MO) was added at 100 ng/mL together with fresh medium containing RvE1. After 6 h of incubation, the supernatants and cells were collected and stored at -80°C. In another set of experiments, HMDMs were incubated at 37°C in a 5% CO_2_ atmosphere in the presence of either vehicle (1:10 DPBS^-/-^ in RPMI) or recombinant human CD5L (catalog # 2797-CL-050, RD systems, Minneapolis, MN) (1 µg/mL) for 20 h and then exposed to LPS (100 ng/m) for 4 additional hours. At the end of the incubation period, the supernatants and cells were collected and stored at -80°C.

### EV Purification

EVs were isolated from plasma by Size Exclusion Chromatography (SEC) as described before (13). Briefly, 1.5 mL of plasma was centrifuged at 2000 g for 1 min at 4°C and then loaded into 20 mL of Sepharose-CL2B (Sigma-Aldrich) previously packed into a Puriflash Dry Load Empty column (Interchim, France). Sample separation and elution were performed using filtered PBS. Thirty-five fractions of 0.5 mL were collected. Fractions 5 to 14 were analyzed for the expression of the tetraspanin-specific EV marker CD9 and CD5L by bead-based flow cytometry. Briefly, EVs were coupled to 4 µm aldehyde-sulphate latex beads (Invitrogen Ref A37304) for 15 min. Then, 1 mL of PBS containing 0.1% BSA and 0.01% Sodium Azide (coupling buffer) in PBS was added and EVs were incubated overnight at rt on rotation before incubation with anti-CD9 (RRID : AB_10627954) and anti CD5L (14) mAbs for 30 min at 4°C, followed by anti-mouse IgG-FITC antibody (BD Biosciences, San Jose, CA, ref. 555988, 1:100 dilution) for 30 min at 4°C. Unbound antibodies were washed off with coupling buffer by centrifugation at 2,000 *g* for 10 min between steps. Samples were analyzed using a FACSLyric flow cytometer (BD Biosciences). In this regard, 10,000 single beads per sample were examined, and mean fluorescence intensity (MFI, Flow Jo software, Tree Star, Ashland, OR) was used to compare different fractions. CD9-positive fractions were considered as EV-containing fractions. The 3 fractions containing the highest CD9 MFI were pooled for subsequent Nanoparticle Tracking Analysis (NTA) and cryo-electron microscopy studies. The protein concentration of each fraction was measured by bicinchoninic acid (BCA) assay, following the manufacturer´s instructions (Pierce, Thermo Fisher Scientific, Waltham, MA).

### Western Blotting of EV

1.5 ml of eluted EVs were concentrated 8-fold by centrifugation at 2000 g for 20 min at 4°C, using a 100 kDa cutoff centricon (MerckMillipore, Darmstadt, Germany). THP1 cells, used as positive control, were grown in RPMI containing 10% FCS and P/S (14). Cell lysates were prepared in RIPA buffer (50 mmol/l Tris-HCl pH 8, 150 mmol/l NaCl, 1% Nonidet P-40, 0.1% SDS, 1% Triton X-100 plus proteinase inhibitors). Concentrated EVs or 30 µg of THP1 cell lysates were resolved in 10% SDS-polyacrylamide gels under non-reducing conditions and electrophoretically transferred to nitrocellulose membranes (Bio-Rad Laboratories). These were then blocked with Starting Block TBS buffer (Thermo Fisher) for 1 h at rt and incubated overnight at 4°C with anti-CD9 (Immunostep) or CD63 mAb (RRID : AB_396297) diluted in blocking buffer. The membranes were subsequently incubated for 60 min at rt with IRDye 800Cw-conjugated goat anti-mouse IgG (LI-COR Biosciences Lincoln, NE) (RRID : AB_621842) diluted in blocking buffer. Three 15-min washes between steps were performed with TBS-0.01% Tween 20 (Merck Millipore). Bound antibody was detected with an Odyssey Infrared Imager (LI-COR), and densitometric analysis was performed using the Odyssey V.3 software (LI-COR).

### Nanoparticle Tracking Analysis

Size distribution and concentration of purified EVs (n=4/group) were determined in a NanoSight LM10 instrument (Malvern Instruments Ltd, Malvern, UK) equipped with a 638 nm laser and CCD camera (model F-033), and data were analyzed with the Nanoparticle Tracking Analysis (NTA) software version 3.2 (Build 3.1.46). The detection threshold was set to 5, and blur and Max Jump Distance were set to auto. Samples were diluted 10 or 20 times with PBS to reach the optimal concentration for instrument linearity: 20-120 particles/frame as advised by the manufacturer. Readings were taken on triplicates of 60 s at 30 frames per second (fps), at a camera level set to 16 and with manual monitoring of temperature.

### Cryo-Electron Microscopy

Selected SEC fractions showing the highest MFI for CD9 marker were examined for EV size and morphology by cryo-electron microscopy (cryo-EM). A total of 3 preparations from each group were analyzed. Vitrified specimens were prepared by placing 3 μl of a sample on a Quantifoil^®^ 1.2/1.3 TEM grid, which was blotted to a thin film and plunged into liquid ethane-N2(l) in the Leica EM CPC cryoworkstation (Leica, Wetzlar, Germany). The grids were transferred to a 626 Gatan cryoholder and kept at -179°C. Samples were analyzed with a Jeol JEM 2011 transmission electron microscope (Jeol, Tokyo, Japan) operating at 200 kV. Images were recorded on a Gatan Ultrascan 2000 cooled charge-coupled (CCD) camera with the Digital Micrograph software package (Gatan, Pleasanton, CA). Aliquots of 10 µL of samples were laid on formvar-Carbon EM grids, frozen, and immediately analyzed with a Jeol JEM 2011 transmission electron microscope operating at 200 kV. Samples were kept at -182°C during imaging (626 Gatan cryoholder). Images were recorded on a Gatan Ultrascan CCD camera under low electron dose conditions to minimize electron beam radiation.

### mRNA Expression Analysis

Total RNA was isolated from cells using the TRIzol reagent and following the manufacturer’s instructions. RNA concentrations were assessed in a NanoDrop-1000 spectrophotometer (Thermo Fisher). cDNA synthesis from 200 to 1000 ng of total RNA was performed using the High-Capacity cDNA Archive Kit (Applied Biosystems, Foster City, CA). Real-time PCR analysis of human genes was performed in a 7900HT Fast System (Applied Biosystems) using commercial probes for arachidonate 5-lipoxygenase (5-LOX, Hs00167536_m1), arachidonate 15-lipoxygenases [15-LOX-1, Hs00993765_g1 and 15-LOX-2, type B Hs00153988_m1), arachidonate 12-lipoxygenase, 12S type (12-LOX, Hs00167524)], cytochrome P450 family 2 subfamily J member 2 (CYP2J2, Hs00356035_m1), prostaglandin-endoperoxide synthase 1 (COX-1, Hs00377726_m1), and 2 (COX-2, Hs00153133_m1), using β-actin (ACTB, Hs99999903_m1) as endogenous control. All probes were purchased from Thermo Fisher Scientific (Waltham, MA). Quantitative PCR results were analyzed with Sequence Detector 2.1 Software (Applied Biosystems). For CD5L expression probes (Hs.PT.56a.39293248) and glyceraldehyde 3-phosphate dehydrogenase (GAPDH, Hs.PT.39a.22214836) as endogenous control, were purchased from Integrated DNA Technologies (IDT, Iowa, United States). Relative quantification of gene expression was performed using the comparative CT method. The amount of target gene, normalized to ACTB and relative to a calibrator, was determined by the arithmetic equation 2-ΔΔCt, as described in the comparative CT method.

### CD5L Expression Analyses

For analysis of CD5L expression, PBMCs were isolated from the buffy coats of healthy blood donors [provided by the Blood and Tissue Bank (Barcelona, Spain)] following the institutional standard operating procedures for blood donation and processing, including informed consent, as described previously (15). Briefly, PBMCs were purified by Ficoll-Paque (GE Healthcare) density gradient centrifugation and enriched in PB monocytes by adherence. Cells were allowed to differentiate to HMDMs for 10 days in RPMI-1640 2 mM glutamine (Lonza) supplemented with 10% heat-inactivated human AB serum (Sigma-Aldrich), 10% heat-inactivated fetal bovine serum (FBS, Lonza), 100 U/mL penicillin, and 100 µg/mL streptomycin (Sigma-Aldrich) in 6-well plates at a density of 10^6^ cells/well. Twenty-four hours before the experiments, HMDMs were detached with accutase (Sigma-Aldrich) and placed on Millicell EZ slides (Merck Millipore) (10^5^ cells/well). They were then stimulated for 72 h with RvE1 (10 nM) with or without 10 ng/ml LPS in Vehicle (Veh, 0.007% EtOH), or 40 ng/ml Dexamethasone (Kern pharma), used as positive control, as indicated. For mRNA expression analysis, cells were processed as detailed above. For immunofluorescence, cells were fixed with PBS containing 4% paraformaldehyde (Panreac) and incubated for 24 h at 4°C with mAb anti-CD5L in PBS containing 0.3% Triton X-100 and 10% human AB serum (Sigma-Aldrich). Cells were subsequently incubated for 1 h at rt with Alexa Fluor^®^ 488 F(ab’)2 fragment of goat anti-mouse IgG (Molecular Probes) in PBS containing 0.3% Triton X-100. Between steps, unbound antibodies were removed with three washes with PBS. Finally, nuclei were stained for 10 min at rt with PBS containing 800 nM Hoechst 33,258 solution (Sigma-Aldrich). They were then washed three times with PBS, and coverslips were mounted in Fluoromount media (Sigma-Aldrich) and left at 4°C overnight. The slides were examined under an Axio Observer Z1 DUO LSM 710 confocal system and analyzed with ZEN Black software (Carl Zeiss Microscopy GmbH) and ImageJ software, described previously (16).

### Targeted Lipidomics

The extraction protocol and analysis of bioactive lipid mediators were performed as described by Le Faouder et al. (17), adapted by the Ambiotis SAS (Toulouse, France) standard operating procedure. Briefly, samples of plasma, cell supernatants and purified EVs were mixed with 0.4 mL of ice-cold methanol and held at -80°C for protein precipitation. Samples were then centrifuged and supernatants were collected. After removal of the organic solvent under a stream of nitrogen, samples were suspended in methanol and rapidly acidified to pH 3.5 with HCl. Acidified samples were then loaded into C-18 solid-phase extraction cartridges (Waters, Milford, MA), rapidly neutralized, and eluted with methyl formate. Eluate solvents were evaporated under a stream of nitrogen and residues were suspended in mobile phase for liquid chromatography analyses using an Exion LCAD U-HPLC system coupled to a Sciex QTRAP 6500+ MS-MS system (AB Sciex, Framingham, MA), equipped with an ESI source in negative ion mode.

### Measurement of CD5L Levels by Enzyme-Linked Immunosorbent Assay (ELISA)

CD5L levels were assessed in plasma using an in-house developed sandwich ELISA assay, described in (14).

### Measurement of Plasma Cytokines and Chemokines

Cytokine/chemokine levels were determined in 25 μl of plasma using a multiplexed bead-based immunoassay [Milliplex MAP Human Cytokine/Chemokine Magnetic Bead Panel (Merck Millipore, Darmstadt, Germany)] on a Luminex 100 Bioanalyzer (Luminex Corp., Austin, TX). The readouts were analyzed with the standard version of the Belysa software (Merck Millipore). A five-parameter logistic regression model was used to create standard curves (pg/mL) and to calculate the concentration of each sample.

### Statistical Analysis

Results were expressed as mean ± SEM with the number of individual experiments detailed in the Figure Legends. Normal distribution was assessed by d’Agostino and Pearson normality test. Spearman’s test was used for not normally distributed variables correlations. Correlations were calculated only between variables with more than 6 observations. Statistical evaluation of two groups was performed using Student’s t test or the Mann-Whitney test. A p value of <0.05 was considered statistically significant. All statistical analyses were performed using GraphPad Prism versions 5 and 8.

## Results

### Progression of Chronic Liver Disease Alters the Amount of Plasma EVs and Their CD5L Content

**Figure 1:**
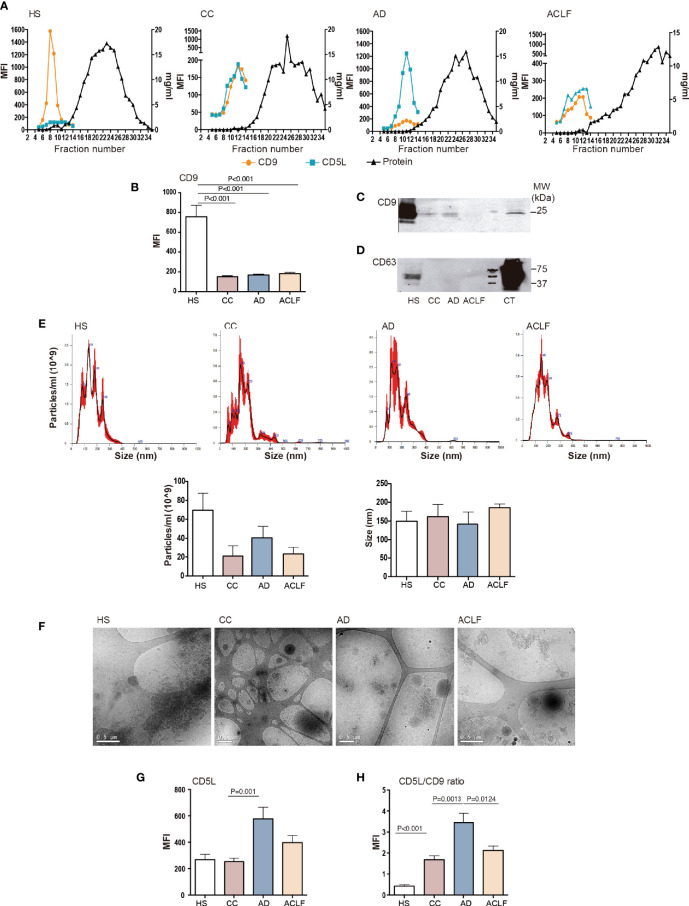
EV characterization. Plasma fractions were pooled to generate n=6 HS, n=6 CC, n=11 AD and n=11 ACLF patient samples**. (A)** Upper panel: Representative graphs showing analysis of SEC eluted fractions. Left axis: Mean Fluorescence intensity (MFI) of EV markers CD9 and CD5L by bead-based flow cytometry. Right axis: protein elution was monitored by BCA. **(B)** CD9 MFI data of all samples analyzed. **(C)** Western blot of CD9 and **(D)** Western blot of CD63 in EVs and THP1 cell lysates, used as positive control (CT). **(E)** Upper panel: NTA of purified EVs from each group of patients. Lower panel: graph depicting particles/ml and size (nm) of the EV particles from 3 purifications. **(F)** Cryo-EM images confirmed EV presence in pooled fractions (F5-7) of SEC preparation from each group of patients. **(G)**. CD5L and **(H)** the ratio of CD5L vs. CD9 MFI data. Bars represent mean ± SEM. P values were calculated using the Mann-Whitney test. HS, healthy subject; CC, Compensated Cirrhotic; AD, Acute Decompensation without ACLF; ACLF, Acute-on-Chronic Liver Failure.

To characterize the CD5L content of EVs, we purified EVs by SEC from total plasma from HS (n=6) and patients with liver cirrhosis at different stages of severity [CC (n=6), AD (n=11) and ACLF (n=11)]. The clinical and laboratory data for these patients are given in **Supplementary Table 1**. **Figure 1A** shows that the elution pattern was similar in all conditions and that EVs were successfully separated from the bulk of protein, as determined by the positivity for the EV-associated tetraspanin CD9 in bead-based flow cytometry and the BCA assay, respectively. These experiments showed that CD9 levels dropped dramatically at all stages of liver disease (CC, AD, ACLF) as compared to HS (**Figure 1B**). Western blotting confirmed reduced CD9 content in EVs from patients with cirrhosis (**Figure 1C**). Likewise, western blotting against the tetraspanin CD63, another constitutive marker of EV, further confirmed this suppression (**Figure 1D**). To further confirm the purification of EVs, a subset of SEC fractions was analyzed by NTA and processed for cryo-EM. NTA indicated the presence of particles with a similar mean size of 152 nm for all groups, and an average of 70 ± 36 particles/ml in HS and a much lower content, namely 20 ± 19, 41 ± 24 and 23 ± 15 particles/ml, in CC, AD and ACLF, respectively (**Figure 1E**). Cryo-EM confirmed that EV fractions contained round-shaped nanovesicles (**Figure 1F**). Of note, CD5L content was similar in EVs purified from HS and CC, while this parameter reached its highest value in AD and decreased in ACLF (**Figure 1G**). These observations can be better appreciated by plotting the MFI signal for CD5L against that for CD9 (CD5 vs. CD9 ratio), which showed a significant increase in CC vs. HS, a further increase in AD vs. CC and a significant reduction in ACLF as compared to AD (**Figure 1H**). Taken together, these results indicate a loss of circulating EVs at all stages of liver cirrhosis and a differential content in CD5L as the disease progressed.

### Chronic Liver Disease Is Associated With Changes in the Profile of Bioactive Lipid Mediators in Plasma EVs

**Figure 2:**
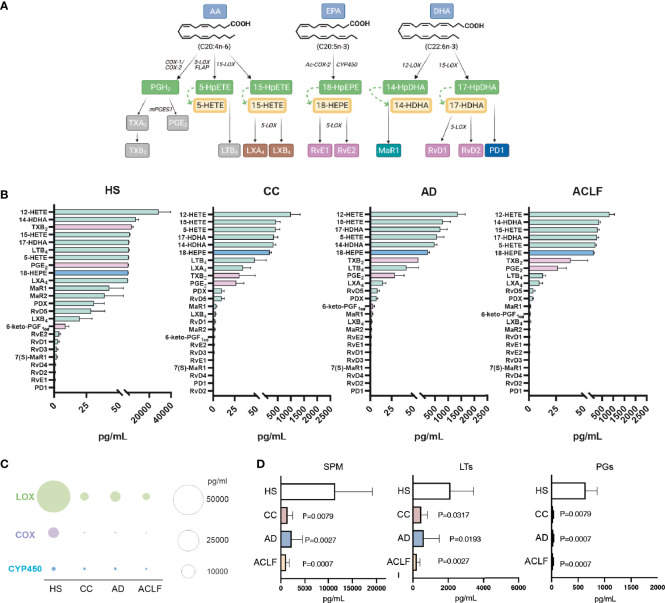
Analysis of eicosanoid and SPM content by liquid chromatography-tandem mass spectrometry (LC-MS/MS)-based lipidomics in EV from HS (n= 5), CC (n=5), AD (n=10) and ACLF (n=10) patient samples. **(A)** Schematic diagram of SPM biosynthesis from arachidonic acid (AA), eicosapentaenoic acid (EPA) and docosahexaenoic acid (DHA). **(B)** Lipid mediators are ranked on the basis of their concentration (the highest on the top; the lowest on the bottom) in the four groups. Color of bars indicates the biosynthetic pathway of the lipid mediators: green-LOX, purple-COX, and blue-Cyp450. **(C, D)**. Total amount of lipid mediators calculated by the sum of the concentrations of each lipid mediator categorized according to the biosynthetic LOX, COX and CYP pathways **(C)**. or to the lipid mediator biochemical family **(D)**. Bars represent mean ± SEM. P values were calculated using the Mann-Whitney test. HS, healthy subject; CC, Compensated Cirrhotic; AD, Acute Decompensation without ACLF; ACLF, Acute-on-Chronic Liver Failure.

Since EVs are a nidus for the formation of bioactive lipid mediators, we next used LC-MS/MS to characterize the profile of lipid mediators in purified EVs. **Figure 2A** shows a schematic diagram of the lipid mediator biosynthesis from arachidonic acid (AA), eicosapentaenoic acid (EPA) and docosahexaenoic acid (DHA). The top five most abundant lipid mediators in EVs from HS were 12-hydroxyeicosatetraenoic acid (12-HETE) (produced by platelets), followed by 14-HDHA, TXB_2_, 15-HETE and 17-HDHA (**Figure 2B**). The list of the top five abundant lipid mediators remained the same in EVs from patients with cirrhosis, except that TXB_2_ was replaced by 5-HETE (**Figure 2B**). Other lipid mediators detected in EVs were LTB_4_, PGE_2_, 18-HEPE (the precursor of resolvins of the E-series), LXA_4_ and LXB_4_ (SPMs derived from 15-HETE), 14-HDHA-related SPMs (maresin [MaR] 1, MaR2 and 7(S)Mar1), resolvin (Rv) D5 (SPM derived from 17-HDHA), 6-keto-PGF_1α_ and a long list of other SPMs, including resolvins of the E- series (i.e. RvE2 and RvE1) and members of the D-series resolvins (RvD1, RvD2, RvD3 and RvD4) and protectins (PD1 and PDX) (**Figure 2B**). We next assigned each lipid mediator to its corresponding biosynthetic enzymatic pathway and identified that LOX-derived products were largely dominating the composition of EVs in patients with cirrhosis (**Figure 2C**). We also assigned each lipid mediator to the corresponding prostaglandin (PG), leukotriene (LT) or SPM family. The list of lipid mediators ascribed to each family is shown in **Supplementary Table 2**. In absolute values, EVs were predominantly enriched in SPMs and SPM intermediate precursors, followed by LTs and finally PGs (**Figure 2D**).

### E-Series Resolvins Are Significantly Decreased in EVs From Patients With AD Cirrhosis That Develop ACLF

**Figure 3:**
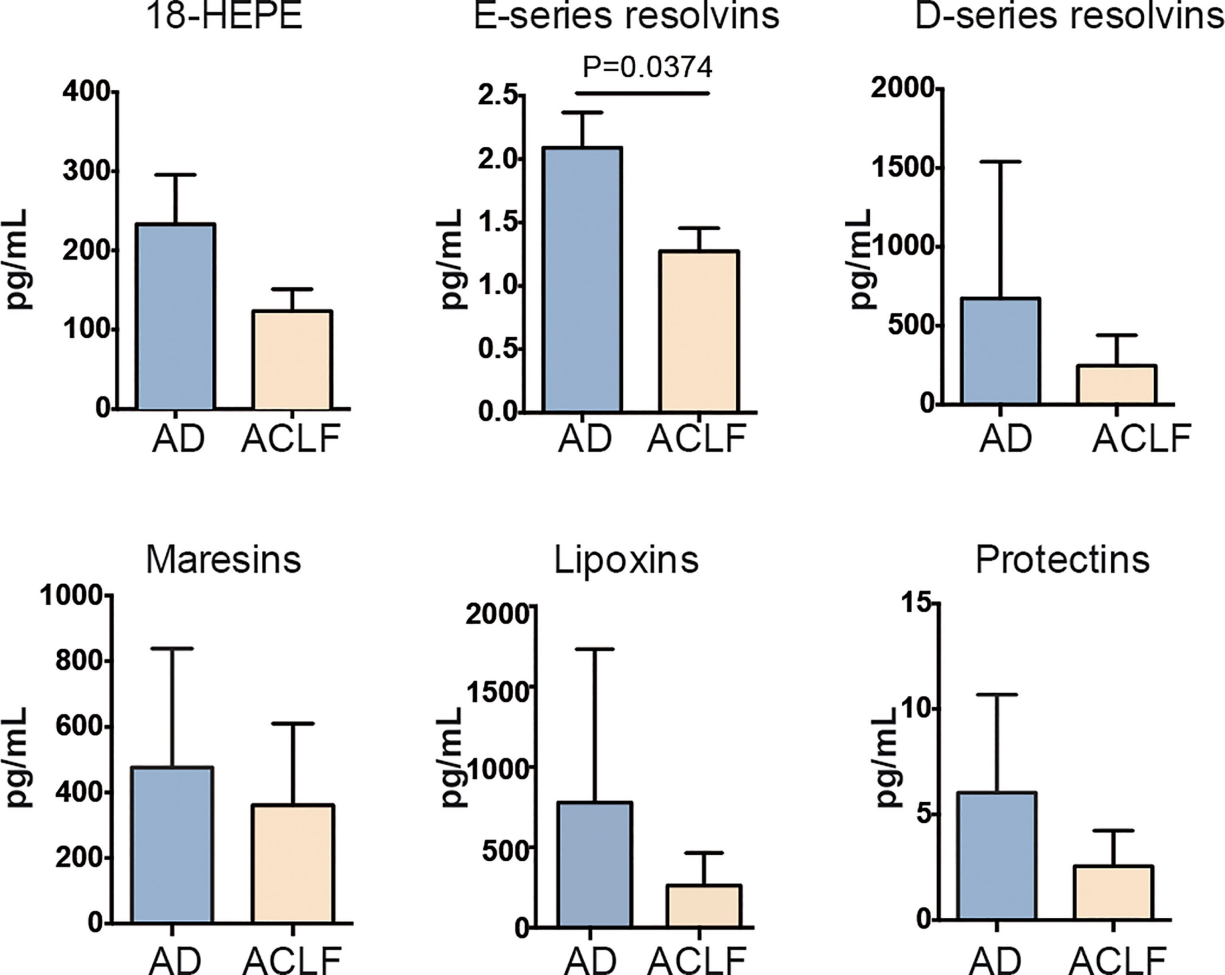
Drop of E-series resolvins in EVs from ACLF patients. Individual SPM EV content in AD vs ACLF patients. Bars represent mean ± SEM. P values were calculated using the Mann-Whitney test. Data from AD (Acute Decompensation without ACLF) (n=10) and ACLF (Acute-on-Chronic Liver Failure) (n=10) patient samples.

Since SPM were preponderant in the composition of lipid mediators in EVs, we then focused on the differences in SPM content in EVs between patients with AD cirrhosis that had developed ACLF and those patients with AD cirrhosis who hadn’t. We categorized each SPM (including the SPM precursors) to the different SPM sub-families (see the list of SPMs ascribed to each sub-family in **Supplementary Table 3**). Interestingly, the EV content of the E-series precursor 18-HEPE was lower in AD patients with ACLF than in those without, and this was consistent with a significant loss of E-series resolvins in ACLF patients (**Figure 3**). This finding indicates that EVs from patients with AD cirrhosis lose their E-series SPM content during the development of ACLF. Furthermore, RvE1 showed the highest correlation with CD5L among the different lipid mediators (**Supplementary Figure 1**). Taken together, these findings suggest that at the most severe stages of liver disease (i.e., ACLF), there is a loss of pro-resolving pathways, which may favor the development of a systemic hyperinflammatory state, leading to organ dysfunction or failure.

### Suppression of the Plasma Levels of CD5L in the Most Severe Stages of Advanced Liver Cirrhosis: Relationship With Extrahepatic Organ Failures

**Figure 4:**
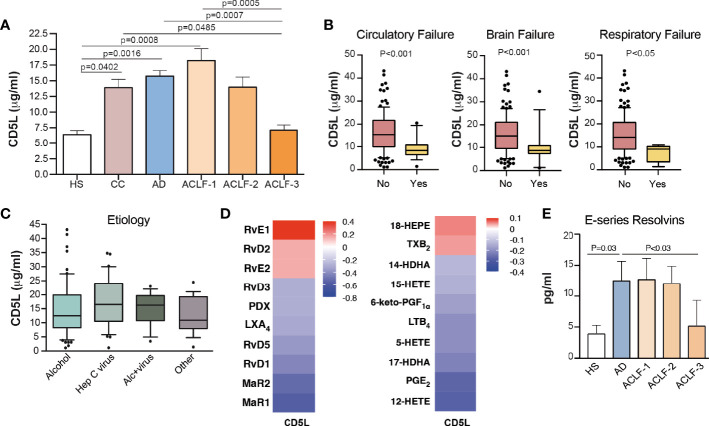
Plasma CD5L levels and RvE1 decrease in ACLF. **(A)** Plasma levels of CD5L were measured by ELISA in HS (n=10), CC (n=20), AD (n=80) and ACLF (n=69) patient samples (ACLF-1 n=33, ACLF-2 n=21, ACLF-3 n=15). Bars indicate the mean values ± SEM for CD5L in each group. **(B)** Association of CD5L with circulatory (n=17), brain (n=17) and respiratory (n=4) failures. **(C)** Association of CD5L with alcohol (n=63), hepatitis C virus (Hep C virus, n=37), alcohol + hepatitis C virus (Alc+virus, n=17) and other (n=18) etiologies. Data represent median plus interquartile range. **(D)** Spearman´s correlation heat map with correlation coefficient between CD5L and lipid mediator concentration, determined by LC-MS/MS in the plasma of AD (n=10) and ACLF (n=15) patients. **(E)** E series Resolvins (RvE1 and RvE2) plasma concentration, determined as in D) and in additional n=5 HS. Bars represent mean ± SEM. D. P values were calculated using the Mann-Whitney test. HS: healthy subject; CC, Compensated Cirrhotic; AD, Acute Decompensation without ACLF; ACLF, Acute-on-Chronic Liver Failure.

We next sought to compare the CD5L content in EVs to that in total plasma in a much larger cohort of patients by using ELISA. In these measurements, we included 10 HS, 20 CC, 80 AD without ACLF and 69 AD patients with ACLF. The baseline clinical and standard laboratory data of the patients included in CD5L measurements are shown in **Supplementary Table 4**. The mean ± SEM plasma CD5L concentration as analyzed by ELISA in HS patients was 6.4 ± 0.6 μg/ml, while this parameter increased steadily in CC and AD patients, peaked in patients with ACLF (grade 1), and then dropped in those with the most severe forms of the disease (ACLF grades 2 and 3), reaching statistical significance in patients with ACLF grade 3 with respect to AD and ACLF grade 1 (**Figure 4A**). Remarkably, significantly lower levels of CD5L were observed in patients with either circulatory, brain or respiratory failure (**Figure 4B**), without apparent changes in their levels in patients with liver, coagulation or renal failure (**Supplementary Figure 2A**). No significant association was found between plasma levels of CD5L and bacterial infection, development of bacterial infection during hospitalization, or 28-day mortality (**Supplementary Figures 2B, C**). Levels of CD5L did not predict the clinical outcome of the patients but plasma levels were lower in those worsening than in those with improved or steady clinical course (mean ± SEM 14.07 ± 2.31 vs 16.08± 1.73 or 15,67 ± 0.96 µg/ml), although differences did not reach statistical significance (**Supplementary Figures 2D, E**). Of note, lower plasma levels of CD5L were present in patients with cirrhosis of alcohol etiology in comparison to those of hepatitis C virus origin, without reaching statistical significance (**Figure 4C**). Together, these findings suggest that development of extrahepatic organ failures (i.e. circulatory, brain or respiratory failures) in patients with well advanced and decompensated liver cirrhosis could be associated with defective circulating CD5L concentrations.

### Translation to the Systemic Circulation of the Defect in CD5L and RvE1 Levels

We next sought to investigate the impact of reduced plasma CD5L levels in the most severe forms of the disease with systemic inflammation. As shown in **Supplementary Figure 3A**, plasma CD5L levels showed a negative correlation with white blood cell (WBC) and platelet counts. In contrast, CD5L levels showed a highly significant positive correlation with IL-1α, IL-10, MCP-1 and IFNα2, and negative correlation with IL-17A (**Supplementary Figure 3B**). Of interest, CD5L significantly positively correlated with IgG concentration (**Supplementary Figure 3C**). We also correlated the CD5L content with the content of lipid mediators measured by LC-MS/MS in total plasma from 5 HS, 10 AD and 15 ACLF patients. This analysis revealed that CD5L strongly correlated with RvE1, as well as with its intermediate precursor 18-HEPE (**Figure 4D**). Additionally, plasma levels of E-series resolvins RvE1 and RvE2 were significantly suppressed in patients afflicted by grade 3 ACLF in comparison to patients with AD (**Figure 4E**). Overall, these data indicate that the pattern of suppression of anti-inflammatory and pro-resolving molecules (CD5L and RvE1) in the plasma of patients with cirrhosis parallels that seen in EVs, and that impairment of these endogenous braking signals is a hallmark in patients presenting the most severe forms of advanced liver cirrhosis.

### Identification of a Positive Feedback Loop Between RvE1 and CD5L in Macrophages

**Figure 5:**
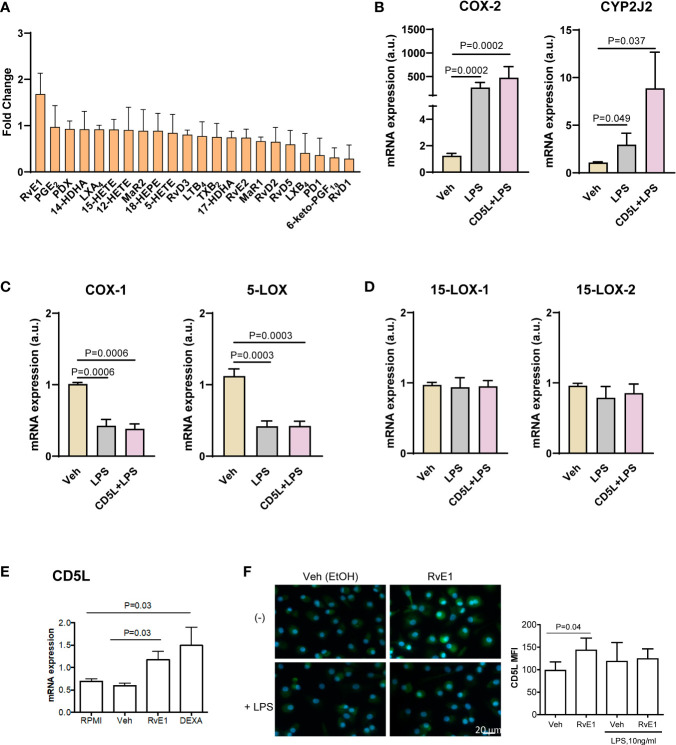
Positive feed-back between CD5L and RvE1 synthesis in macrophages. Peripheral blood mononuclear cells (PBMCs) were isolated from the blood of healthy donors (n=8) by ficoll gradient centrifugation. **(A)** PBMCs were stimulated with 1 μg/ml CD5L or Vehicle (Veh, PBS) for 24 h, and SPMs in the supernatants were quantified by LC-MS/MS. **(B–D)** PBMCs were cultured for 7 days and differentiated into primary macrophages, which were stimulated with 1 μg/ml CD5L for 24 h with or without 100 ng/ml LPS during the last 4 h of culture, and the amount of mRNA encoding the indicated enzymes was quantified by RT-qPCR. **(E, F)** Human primary macrophages (n=5 donors) were treated for 72h with RvE1 (10 nM) with or without 10 ng/ml LPS in Vehicle (Veh, 0.007% EtOH), or 40 ng/ml Dexamethasone (DEXA), used as positive control. **(E)** The amount of mRNA encoding CD5L was measured by RT-qPCR. **(F)** Representative immunofluorescence images of CD5L (green) in primary treated Nuclei were stained with Hoechst (blue). Graphs show CD5L mean fluorescence intensity (MFI) ± SEM of 50 macrophages scored in random fields. Bars represent mean ± SEM. P values were calculated using the Mann-Whitney test.

To study whether there is a direct link and/or a causal relationship between CD5L and the biosynthesis of RvE1, we next carried out mechanistic studies in peripheral blood mononuclear cells (PBMCs) and macrophages derived from circulating peripheral blood monocytes from healthy donors (human monocyte-derived macrophages, HMDM). Given that HMDMs in culture express no detectable levels of CD5L protein (16, 18), we supplemented these cell cultures with recombinant human CD5L (rCD5L) and collected the supernatants to determine the levels of lipid mediators by LC-MS/MS. We also collected the cell extracts to assess the effects of rCD5L on the expression of genes involved in the SPM biosynthetic pathways in the presence of LPS (to mimic an inflammatory milieu). **Figure 5A** shows the effects of rCD5L on all the SPMs detected in the PBMCs supernatants. Unlike the AA-derived eicosanoids and the DHA-derived D-series resolvins, protectins and maresins, rCD5L induced a ~1.5-fold increase in the EPA-derived SPM RvE1. Consistent with enhanced RvE1 formation, in the presence of LPS, rCD5L upregulated the expression of COX-2 and CYP2J2 (**Figure 5B**), the two enzymes involved in the initial stages of RvE1 biosynthesis from EPA. rCD5L did not modify the LPS-induced downregulation of constitutive enzymes such as COX-1 and 5-LOX (**Figure 5C**) and did not affect the expression of 15-LOX-1 and -2 (**Figure 5D**). Conversely, when HMDMs were exposed to RvE1, the expression of CD5L mRNA and protein was increased, as observed by RT-qPCR (**Figure 5E**) and immunofluorescence microscopy using an anti-CD5L mAb (**Figure 5F**). Taken together, these data point to positive feedback between CD5L and the RvE1 axis in human macrophages.

## Discussion

Here we isolated and characterized EVs from the plasma of patients at different stages of liver disease, with a focus on ACLF. Given the alterations in EV composition in liver pathology, these structures have been proposed as a source of biomarkers, as well as potential tools for therapeutic intervention (12). Our results support this notion since we show that circulating CD5L and EV content of lipid mediators are not only altered in cirrhosis vs. healthy subjects but also differ between disease stages, namely compensated vs. decompensated with or without ACLF.

Previous findings suggest an increase in circulating liver-derived micro-vesicles (i.e., plasma cell-derived EVs) in cirrhosis (19–23). Additionally, it has been demonstrated that stressed hepatocytes in culture enhance the production of EVs (19), and a study showed that more EVs carrying ASPGRP hepatocyte marker are detected in SEC-purified serum of cirrhotic NASH patients than in healthy subjects (24). In contrast to these data, we observed a loss of circulating EVs in total plasma in compensated and also in decompensated cirrhosis. EV research is evolving rapidly, and various biomarkers and a range of purification methods may result in a different interpretation of the data. In this publication, it was agreed that SEC, the purification approach we used, is low recovery but specific method of purification, according to the Minimal information for studies of extracellular vesicles 2018 (25). More importantly, in our study, we did not aim to analyze hepatocyte-derived EVs but rather the global status of circulating EVs, since our focus was ACLF in decompensated cirrhosis and not liver centered. Although the central organ in these patients is the deteriorated liver, numerous organs or system dysfunctions are involved, such as renal, brain, circulatory, coagulation, respiratory and immune system, among others (26). Therefore, we analyzed circulating EVs regardless of their organ of origin and observed a significant loss of the number of plasma EVs in patients with cirrhosis and at advanced stages of the disease, as reflected by the lower levels of EV markers CD9 and CD63, and the number of particles indicated by NTA technology.

A limitation of our study is that we did not analyze markers of subcellular origin (e.g. phosphatidylserine as a marker for apoptotic bodies and Grp94 as ER marker) to understand the distribution and entity of purified EVs. Nevertheless, our main interest was the composition of isolated EVs and whether this parameter could provide insight into the possible physiological relationship between CD5L and lipid mediators. The results revealed that CD5L is present in EVs purified from plasma, in agreement with previous reports (5, 27). Interestingly, our data show that the EV cargo of CD5L undergoes significant changes during the course of liver pathology, thereby suggesting that CD5L is not a constitutive biomarker of circulating EVs. Of note, CD5L located in the EV fraction accounted for a very small portion of total plasma CD5L content (data not shown).

Overall, our results reinforce the notion that circulating CD5L (in EVs and total plasma) is strongly associated with the severity of liver disease (14, 28–30). Regarding ACLF, we report for the first time a loss of circulating CD5L in this condition and a significant association with circulatory, brain and respiratory failure. The precise molecular events involved in this association are currently unknown. In contrast, CD5L systemic levels were not modified in bacterial infection, although the latter is associated with substantial morbidity and mortality in these patients. Previous studies have described that CD5L interacts with bacteria, thus pointing to a functional link with infection (31, 32). The heterogeneity of infectious agents in this cohort of patients may preclude the observation of systemic changes in CD5L related to infection. In blood, CD5L can circulate in its free form, in EVs, and in association with IgM but not IgG (33–35). In obesity, this IgM-CD5L association has a functional consequence, since it contributes to auto-antibody production (35). Little is known about its involvement in other contexts. However, in healthy individuals and in cirrhotic patients with liver cancer, IgM and CD5L levels are positively correlated (35, 36). In contrast, we found that CD5L was positively associated with IgG but not with IgM or IgA in AD cirrhosis and ACLF. Our results suggest that deregulation of immunoglobulin homeostasis in ACLF also affects the IgM-CD5L ratio. How CD5L distributes within the three compartments (i.e., free, EVs or IgM) in ACLF and the functional consequences of this distribution remains to be elucidated.

On the other hand, CD5L was found to be strongly positively correlated with IFNα2 and MCP1, two inflammatory mediators. In this regard, patients presenting ACLF have been reported to show significantly higher levels of proinflammatory cytokines/chemokines, including TNF-α, MCP-1, IL-8, and IFN-γ, and also anti-inflammatory IL-10 than patients without ACLF (37). Further studies are needed to unravel the relationship between CD5L and these specific cytokines/chemokines in systemic inflammation. We previously hypothesized that the increase in CD5L during liver disease may be an adaptive response to liver damage and fibrosis that seeks to counteract inflammatory signaling (14). In ACLF, the loss of regulatory CD5L may contribute to failure to regulate inflammation. Additionally, a drop in SPMs would reduce the capacity to repair and regenerate damaged tissues.

Here we report that loss of SPMs and CD5L in EVs and total plasma may have a causal link, since CD5L and RvE1 are mutually upregulated in macrophages. The relationship between CD5L and lipid homeostasis is consistent with the findings of previous studies that showed that CD5L expression in macrophages is regulated by Liver X Receptor (LXR) (3, 38, 39), a transcription factor activated by oxysterols and specific intermediates in the cholesterol biosynthetic pathway that belongs to the nuclear receptor family (40). Moreover, in Th17 cells, CD5L modulates the processing of PUFAs and the formation of bioactive lipid mediators derived from these fatty acids, mainly evolutionarily conserved SPMs (7).

Liver macrophages are key players in most, if not all, inflammation-related liver disorders, including cirrhosis, because they respond to a wide array of activating signals. They are also capable of clearing apoptotic cells and debris, thus initiating the resolution of inflammation, tissue repair and regeneration. It is well known that SPMs play central roles in promoting these processes (41). Our results now suggest that mutual regulation of CD5L and RvE1 provides a novel link between innate immunity and lipid homeostasis. This finding may be highly relevant for understanding how macrophages regulate inflammation and its resolution.

## Data Availability Statement

The datasets presented in this article are not readily available because human sample clinical data will not be shared. Requests to access the datasets should be directed to M-R Sarrias, mrsarrias@igtp.cat; Joan Clària, jclaria@clinic.cat.

## Ethics Statement

The studies involving human participants were reviewed and approved by Human Ethics Committee of the Hospital Clínic of Barcelona; Human Ethics Committee of the Hospital Germans Trias i Pujol. The patients/participants provided their written informed consent to participate in this study.

## Author Contributions

MBS-R, ET, and MC collected, analyzed and interpreted the data. FB and VA performed data analysis and interpretation. JC and M-RS contributed equally to literature search, study design, data analysis and interpretation, and writing. All authors contributed to the article and approved the submitted version.

## Funding

This study was supported by the EF Clif, European Foundation for the study of chronic liver failure, a non-profit organization that receives unrestricted donations from the Cellex Foundation, Grifols and the European Union’s Horizon 2020 research and innovation program (grant agreements 825694 and 847949). Additional funders were Instituto de Salud Carlos III (ISCIII), and ERDFs from the EU, ‘Una manera de hacer Europa’, PI19/00523 to M-RS and Ministerio de Ciencia e Innovacion (PID2019-105240RB-I00) to JC. The Innate Immunity lab is accredited as an Emergent Research group by the Catalan Agency for Management of University and Research Grants (2017-SGR-49). M-RS is a researcher from IGTP, a member of the CERCA network of institutes supported by the Health Department of the Government of Catalonia. JC laboratory at the Center Esther Koplowitz, IDIBAPS, is part of the CERCA Programme/Generalitat de Catalunya and is a Consolidated Research Group recognized by the Generalitat de Catalunya (2017SGR1449).

## Conflict of Interest

A patent protecting monoclonal antibodies used herein for ELISA studies has been submitted to the European Patent Office.

## Publisher’s Note

All claims expressed in this article are solely those of the authors and do not necessarily represent those of their affiliated organizations, or those of the publisher, the editors and the reviewers. Any product that may be evaluated in this article, or claim that may be made by its manufacturer, is not guaranteed or endorsed by the publisher.

## Supplementary Material

Supplementary Figure 1 Spearman´s correlation heat map with correlation coefficient between CD5L and lipid mediator concentration in EVs, determined by LC-MS/MS in AD (n=10) and ACLF (n=15) patients pooled plasma.

Supplementary Figure 2
**(A)**. Association of CD5L plasma levels with clinical parameters: **(A)**. Organ failure: liver Yes n=34, No n=107, coagulation Yes n=20, No n=121, renal Yes n=37, No n=104. **(B)**. Bacterial infection: Yes n=15, No n=133, and development of bacterial infection during hospitalization: Yes n=44, No n=96. **(C)**. 28-day mortality: Yes n=31, No n=118. **(D)**. Disease course at 3-day follow-up: Steady n= 89, Improvement n=26, Worsening n=13. Data represent median plus interquartile range. AD steady course: no ACLF during follow-up. ACLF steady course: no change of ACLF grade. Improvement of ACLF: decrease of ACLF by at least one grade during follow-up. Worsening of AD or ACLF: development of ACLF or increase of ACLF by at least one grade during follow-up **(F)**. Table showing the distribution of patients analyzed for disease course according to ACLF grade at inclusion vs ACLF grade follow-up.

Supplementary Figure 3 Correlation of plasma levels of CD5L in AD and ACLF with **(A)**. White blood cell (WBC), platelet counts and C-reactive protein. **(B)**. Circulating cytokines and chemokines. **(C)**. IgG concentration.
