# Supplementary figures and images for: Reduced Plasma Extracellular Vesicle CD5L Content in Patients With Acute-On-Chronic Liver Failure: Interplay With Specialized Pro-Resolving Lipid Mediators
Source: Front Immunol. 2022 Mar 7;13:842996. doi: 10.3389/fimmu.2022.842996 (PMC8940329; doi:10.3389/fimmu.2022.842996)

Supplementary Figure 1

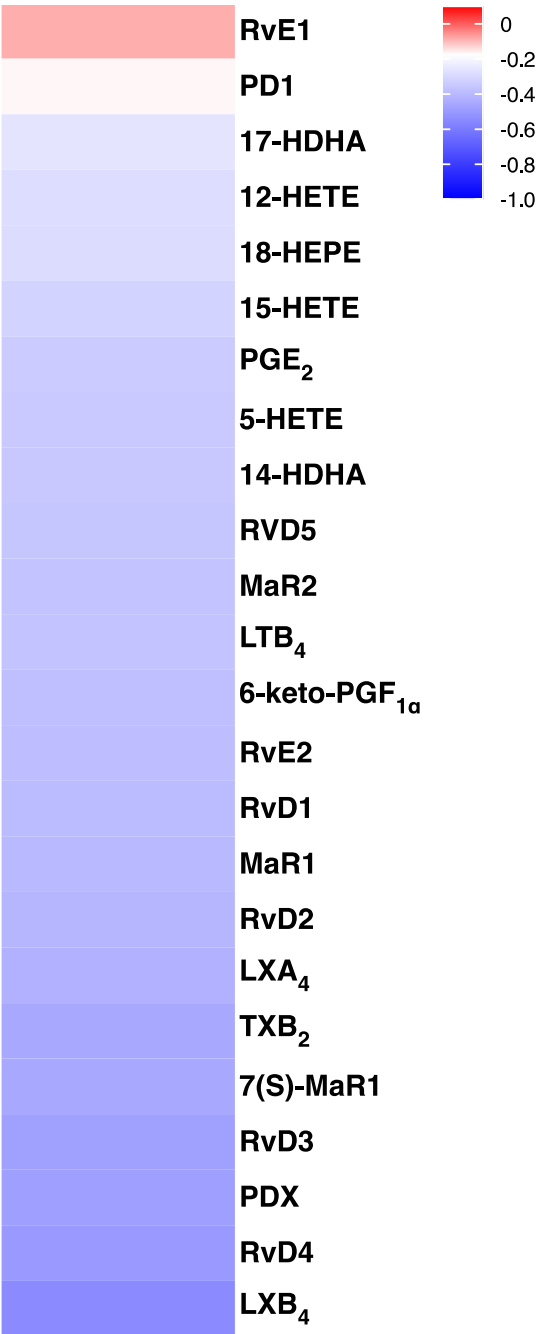

Supplement: Supplementary Figure 1 — Spearman´s correlation heat map with correlation coefficient between CD5L and lipid mediator concentration in EVs, determined by LC-MS/MS in AD (n=10) and ACLF (n=15) patients pooled plasma. [file DataSheet_1.pdf]

Supplementary Figure 2

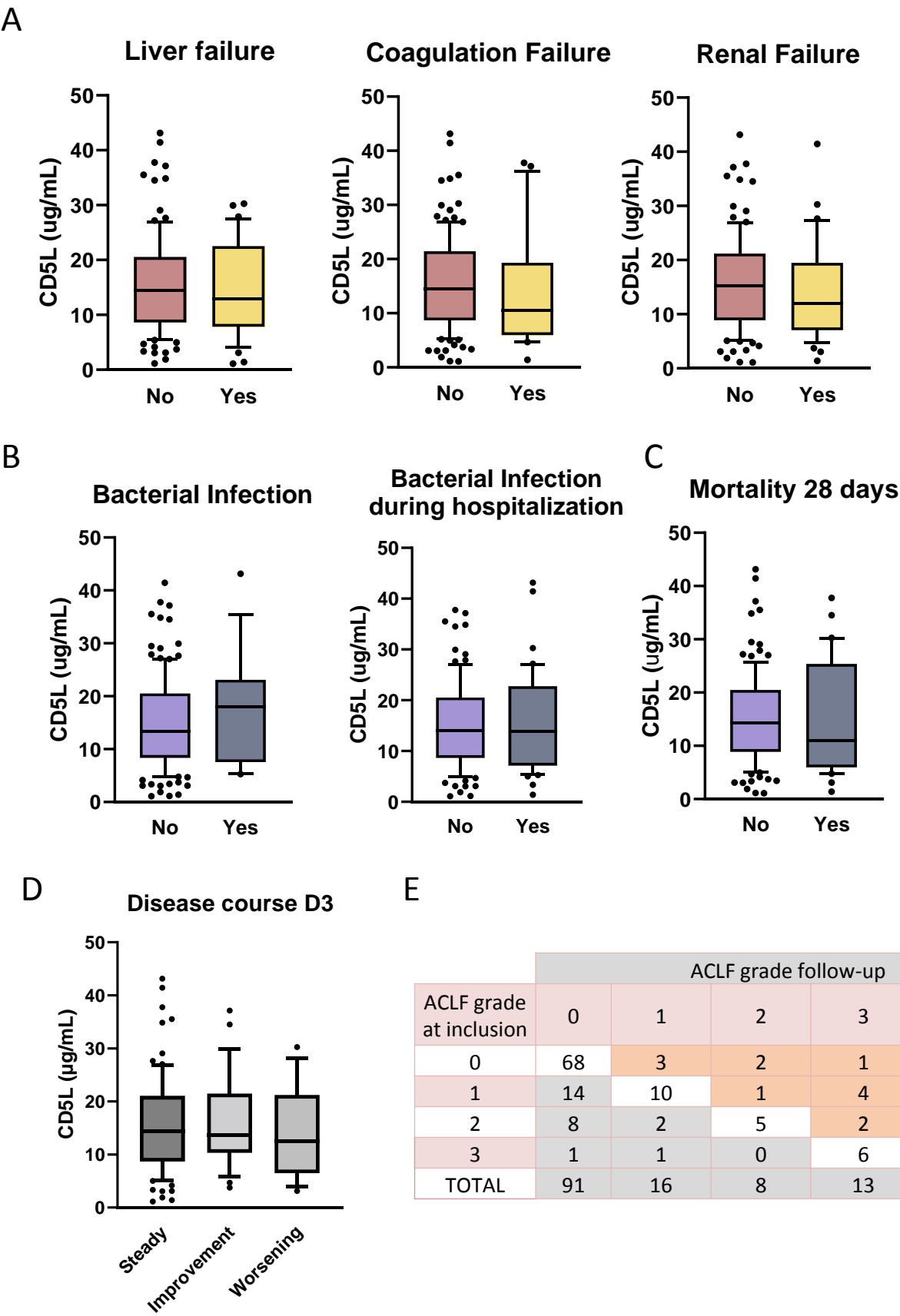

Supplement: Supplementary Figure 2 — (A). Association of CD5L plasma levels with clinical parameters: (A). Organ failure: liver Yes n=34, No n=107, coagulation Yes n=20, No n=121, renal Yes n=37, No n=104. (B). Bacterial infection: Yes n=15, No n=133, and development of bacterial infection during hospitalization: Yes n=44, No n=96. (C). 28-day mortality: Yes n=31, No n=118. (D). Disease course at 3-day follow-up: Steady n= 89, Improvement n=26, Worsening n=13. Data represent median plus interquartile range. AD steady course: no ACLF during follow-up. ACLF steady course: no change of ACLF grade. Improvement of ACLF: decrease of ACLF by at least one grade during follow-up. Worsening of AD or ACLF: development of ACLF or increase of ACLF by at least one grade during follow-up (F). Table showing the distribution of patients analyzed for disease course according to ACLF grade at inclusion vs ACLF grade follow-up. [file DataSheet_2.pdf]

# Supplementary Figure 3

A

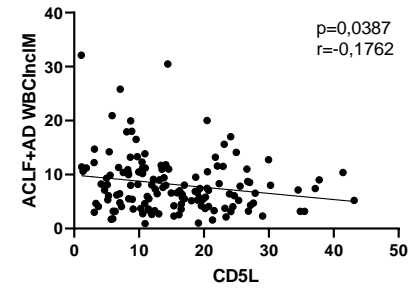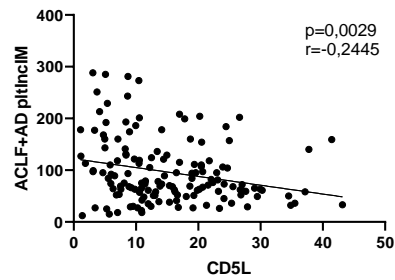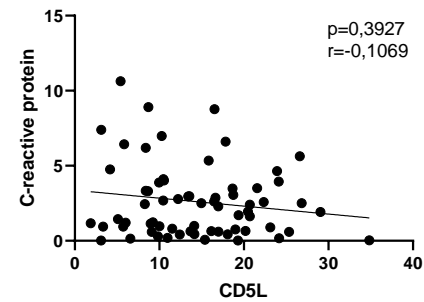

B

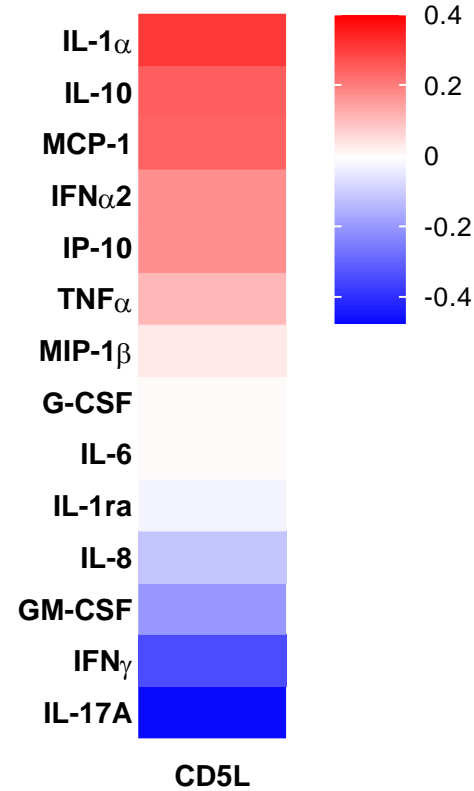

C

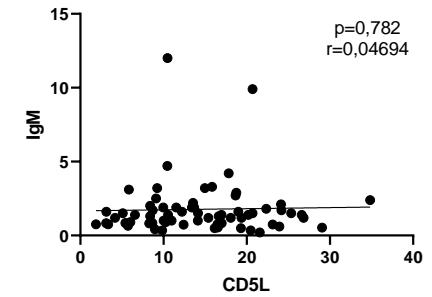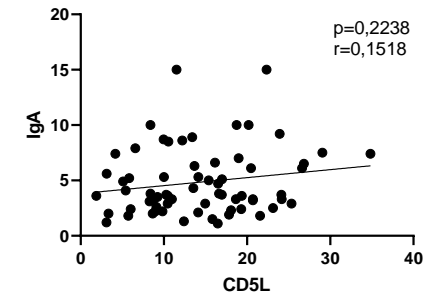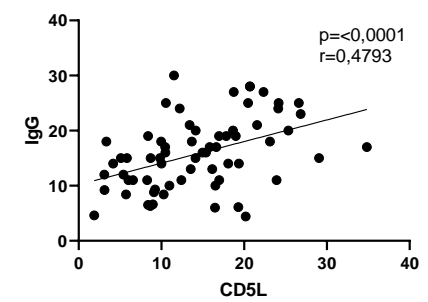

Supplement: Supplementary Figure 3 — Correlation of plasma levels of CD5L in AD and ACLF with (A). White blood cell (WBC), platelet counts and C-reactive protein. (B). Circulating cytokines and chemokines. (C). IgG concentration. [file DataSheet_3.pdf]
