# Supplementary material for: Reduced Plasma Extracellular Vesicle CD5L Content in Patients With Acute-On-Chronic Liver Failure: Interplay With Specialized Pro-Resolving Lipid Mediators
Source: Front Immunol. 2022 Mar 7;13:842996. doi: 10.3389/fimmu.2022.842996 (PMC8940329; doi:10.3389/fimmu.2022.842996)
Supplement: Supplementary file 4 [file Table_1.docx]

**Supplementary Table 1**. Baseline clinical and standard laboratory data of patients with AD cirrhosis and patients with ACLF included in the isolation of extracellular vesicles.

| Variable | AD | ACLF | P value |
| --- | --- | --- | --- |
| Gender (% male) | 68 (53.45) | 53 (41.73) | 0.38 |
| Age (years) | 54.59 +/- 12.42 | 58.19 +/- 11.24 | 0.17 |
| WBC count (x10^9^/L) | 6.41 +/- 2.85 | 9.56 +/- 6.65 | <0.001 |
| Platelet count (x10^9^/L) | 86.31 +/- 52.12 | 91.10 +/- 57.23 | 0.82 |
| CRP (mg/dL) | 28.72 +/- 32.61 | 38.78 +/- 29.14 | <0.01 |
| INR | 1.69 +/- 0.49 | 1.84 +/- 0.61 | 0.29 |
| Serum albumin (mg/dL) | 2.85 +/- 0.52 | 2.92 +/- 0.63 | 0.3 |
| Serum bilirubin (mg/dL) | 5.16 +/- 4.49 | 11.37 +/- 12.70 | 0.07 |
| Serum creatinine (mg/dL) | 1.05 +/- 0.43 | 2.07 +/- 1.39 | <0.001 |
| MELD | 18.33 +/- 5.76 | 25.60 +/- 7.05 | <0.001 |
| CLIF organ failure score | 7.34 +/- 1.19 | 9.95 +/- 1.96 | <0.001 |
| CLIF consortium AD score | 52.33 +/- 7.72 |  |  |
| CLIF consortium ACLF score |  | 48.73 +/- 7.90 |  |
| Child-Pugh score | 9.72 +/- 1.95 | 10.77 +/- 2.16 | <0.01 |
| 28-day mortality | 4 (3.15) | 21 (16.54) | <0.001 |

*Discrete variables are shown as absolute frequencies (percentage) and continuous variables as mean (SD). WBC: White blood cells; CRP: C-reactive protein; INR: international normalized ratio; MELD: model for end-stage liver disease.*
