# Supplementary material for: Reduced Plasma Extracellular Vesicle CD5L Content in Patients With Acute-On-Chronic Liver Failure: Interplay With Specialized Pro-Resolving Lipid Mediators
Source: Front Immunol. 2022 Mar 7;13:842996. doi: 10.3389/fimmu.2022.842996 (PMC8940329; doi:10.3389/fimmu.2022.842996)
Supplement: Supplementary file 5 [file Table_2.pdf]

Supplementary Table 2. List of lipid mediators ascribed to each family.

Family	Common Name	Systematic Name	Main Class	Sub Class
PGs	6-keto-$PGF_{1\alpha}$	6-oxo-9S,11R,15S-trihydroxy-13E-prostenoic acid	Eicosanoids [FA03]	Prostaglandins [FA0301]
	PGE_2	9-oxo-11R,15S-dihydroxy-5Z,13E-prostadienoic acid	Eicosanoids [FA03]	Prostaglandins [FA0301]
	TXB_2	9S,11,15S-trihydroxy-thromboxa-5Z,13E-dien-1-oic acid	Eicosanoids [FA03]	Thromboxanes [FA0303]
LT	5-HETE	5-hydroxy-6E,8Z,11Z,14Z-eicosatetraenoic acid	Eicosanoids [FA03]	Hydroxy/hydroperoxyeicosatetraenoic acids [FA0306]
	12-HETE	12-hydroxy-5Z,8Z,10E,14Z-eicosatetraenoic acid	Eicosanoids [FA03]	Hydroxy/hydroperoxyeicosatetraenoic acids [FA0306]
	LTB_4	5S,12R-dihydroxy-6Z,8E,10E,14Z-eicosatetraenoic acid	Eicosanoids [FA03]	Leukotrienes [FA0302]
SPM	(+/-)-17-HDHA	(+/-)-17-hydroxy-4Z,7Z,10Z,13Z,15E,19Z-docosahexaenoic acid	Docosanoids [FA04]	Other Docosanoids [FA0400]
	(+/-)-14-HDHA	(+/-)-14-hydroxy-4Z,7Z,10Z,12E,16Z,19Z-docosahexaenoic acid	Docosanoids [FA04]	Other Docosanoids [FA0400]
	15-HETE	15-hydroxy-5Z,8Z,11Z,13E-eicosatetraenoic acid	Eicosanoids [FA03]	Hydroxy/hydroperoxyeicosatetraenoic acids [FA0306]
	(+/-)-18-HEPE	(+/-)-18-hydroxy-5Z,8Z,11Z,14Z,16E-eicosapentaenoic acid	Eicosanoids [FA03]	Hydroxy/hydroperoxyeicosapentaenoic acids [FA0307]

Source: LIPID MAPS website
