# Supplementary material for: Reduced Plasma Extracellular Vesicle CD5L Content in Patients With Acute-On-Chronic Liver Failure: Interplay With Specialized Pro-Resolving Lipid Mediators
Source: Front Immunol. 2022 Mar 7;13:842996. doi: 10.3389/fimmu.2022.842996 (PMC8940329; doi:10.3389/fimmu.2022.842996)
Supplement: Supplementary file 6 [file Table_3.docx]

**Supplementary Table 3**. List of SPMs ascribed to each family.

| SPM families | Common Name | Systematic Name | Main Class | Sub Class |
| --- | --- | --- | --- | --- |
| D-series | (+/-)-17-HDHA | (+/-)-17-hydroxy-4Z,7Z,10Z,13Z,15E,19Z- docosahexaenoic acid | Docosanoids [FA04] | Other Docosanoids [FA0400] |
|  | RvD1 | 7S,8R,17S-trihydroxy-4Z,9E,11E,13Z,15E, 19Z-docosahexaenoic acid | Docosanoids [FA04] | Resolvin Ds [FA0403] |
|  | RvD2 | 7S,16R,17S-trihydroxy-4Z,8E,10Z,12E, 14E,19Z-docosahexaenoic acid | Docosanoids [FA04] | Resolvin Ds [FA0403] |
|  | RvD3 | 4S,10,17S-trihydroxy-5E,7E,9E,13Z,15E, 19Z-docosahexaenoic acid | Docosanoids [FA04] | Resolvin Ds [FA0403] |
|  | RvD4 | 4S,5,17S-trihydroxy-6E,8E,10E,13E,15Z, 19Z-docosahexaenoic acid | Docosanoids [FA04] | Resolvin Ds [FA0403] |
|  | RvD5 | 7S,17S-dihydroxy-4Z,8E,10Z,13Z,15E,19Z- docosahexaenoic acid | Docosanoids [FA04] | Resolvin Ds [FA0403] |
| E-series | (+/-)-18-HEPE | (+/-)-18-hydroxy-5Z,8Z,11Z,14Z,16E- eicosapentaenoic acid | Eicosanoids [FA03] | Hydroxy/hydroperoxyeicosapentaenoic acids [FA0307] |
|  | RvE1 | 5S,12R,18R-trihydroxy-6Z,8E,10E,14Z, 16E-eicosapentaenoic acid | Eicosanoids [FA03] | Resolvin Es [FA0314] |
|  | RvE2 | 5S,18R-dihydroxy-6E,8Z,11Z,14Z,16E- eicosapentaenoic acid | Eicosanoids [FA03] | Resolvin Es [FA0314] |
| Maresins | (+/-)-14-HDHA | (+/-)-14-hydroxy-4Z,7Z,10Z,12E,16Z,19Z- docosahexaenoic acid | Docosanoids [FA04] | Other Docosanoids [FA0400] |
|  | MaR1 | 7R,14S-dihydroxy-4Z,8E,10E,12Z,16Z,19Z- docosahexaenoic acid | Docosanoids [FA04] | Maresins [FA0405] |
|  | 7(S)-MaR1 | 7S,14S-dihydroxy-4Z,8E,10E,12E,16Z,19Z- docosahexaenoic acid | Docosanoids [FA04] | Maresins [FA0405] |
|  | MaR2 | 13R,14S-dihydroxy-4Z,7Z,9E,11E,16Z,19Z- docosahexaenoic acid | Docosanoids [FA04] | Maresins [FA0405] |
| Lipoxins | 15-HETE | 15-hydroxy-5Z,8Z,11Z,13E- eicosatetraenoic acid | Eicosanoids [FA03] | Hydroxy/hydroperoxyeicosatetraenoic acids [FA0306] |
|  | LXB4 | 5S,14R,15S-trihydroxy-6E,8Z,10E,12E- eicosatetraenoic acid | Eicosanoids [FA03] | Lipoxins [FA0304] |
|  | LXA4 | 5S,6R,15S-trihydroxy-7E,9E,11Z,13E- eicosatetraenoic acid | Eicosanoids [FA03] | Lipoxins [FA0304] |
| Protectins | PDX | 10S,17S-dihydroxy-4Z,7Z,11E,13Z,15E, 19Z-docosahexaenoic acid | Docosanoids [FA04] | Protectins [FA0404] |
|  | PD1 | 10R,17S-dihydroxy-4Z,7Z,11E,13E,15Z, 19Z-docosahexaenoic acid | Docosanoids [FA04] | Protectins [FA0404] |

Source: LIPID MAPS website
