# Supplementary material for: Reduced Plasma Extracellular Vesicle CD5L Content in Patients With Acute-On-Chronic Liver Failure: Interplay With Specialized Pro-Resolving Lipid Mediators
Source: Front Immunol. 2022 Mar 7;13:842996. doi: 10.3389/fimmu.2022.842996 (PMC8940329; doi:10.3389/fimmu.2022.842996)
Supplement: Supplementary file 7 [file Table_4.docx]

**Supplementary Table 4**. Baseline clinical and standard laboratory data of patients with AD cirrhosis and ACLF included in the plasma assessment of CD5L levels.

| Variable | AD | ACLF | P value |
| --- | --- | --- | --- |
| Gender (% male) | 48 (61.54) | 34 (49.28) | 0.27 |
| Age (years) | 57.56 +/- 11.45 | 57.82 +/- 10.81 | 0.83 |
| WBC count (x10^9^/L) | 7.07 +/- 3.64 | 8.60 +/- 5.39 | 0.17 |
| Platelet count (x10^9^/L) | 99.53 +/- 60.70 | 92.77 +/- 59.48 | 0.46 |
| CRP (mg/dL) | 27.93 +/- 38.19 | 37.58 +/- 33.37 | <0.05 |
| INR | 1.55 +/- 0.31 | 2.06 +/- 0.90 | <0.01 |
| Serum albumin (mg/dL) | 2.90 +/- 0.53 | 2.91 +/- 0.68 | 0.71 |
| Serum bilirubin (mg/dL) | 5.34 +/- 6.57 | 11.33 +/- 12.73 | <0.05 |
| Serum creatinine (mg/dL) | 1.03 +/- 0.38 | 2.34 +/- 1.40 | <0.001 |
| MELD | 17.14 +/- 5.44 | 26.74 +/- 6.94 | <0.001 |
| CLIF organ failure score | 7.02 +/- 1.17 | 10.12 +/- 2.09 | <0.001 |
| CLIF consortium AD score | 52.65 +/- 8.52 |  |  |
| CLIF consortium ACLF score |  | 48.50 +/- 7.59 |  |
| Child-Pugh score | 9.20 +/- 1.93 | 10.28 +/- 2.36 | <0.05 |
| 28-day mortality | 4 (5.13) | 20 (28.99) | <0.001 |

*Discrete variables are shown as absolute frequencies (percentage) and continuous variables as mean (SD). WBC: White blood cells; CRP: C-reactive protein; INR: international normalized ratio; MELD: model for end-stage liver disease.*
